# Synthesis of novel antibacterial and biocompatible polymer nanocomposite based on polysaccharide gum hydrogels

**DOI:** 10.1038/s41598-023-42146-6

**Published:** 2023-10-05

**Authors:** Sherzod Shukhratovich Abdullaev, Raed H. Althomali, Ebraheem Abdu Musad Saleh, Magizov Rustem Robertovich, I. B. Sapaev, Rosario Mireya Romero-Parra, Hashem O. Alsaab, M. Abdulfadhil Gatea, Mohammed N. Fenjan

**Affiliations:** 1 Faculty of Chemical Engineering, New Uzbekistan University, Tashkent, Uzbekistan; 2 Scientific Department, Tashkent State Pedagogical University Named After Nizami, Tashkent, Uzbekistan; 3 Department of Chemistry, College of Arts and Science, Prince Sattam Bin Abdulaziz University, 11991 Wadi Al-Dawasir, Saudi Arabia; 4 Kazan Federal University, Naberezhnye Chelny, Russia; 5 Tashkent Institute of Irrigation and Agricultural Mechanization Engineers, National Research University, Tashkent, Uzbekistan; 6 New Uzbekistan University, Tashkent, Uzbekistan; 7 Universidad Continental, Lima, Perú; 8 Department of Pharmaceutics and Pharmaceutical Technology, Taif University, Taif, Saudi Arabia; 9 Technical Engineering Department College of Technical Engineering, The Islamic University, Najaf, Iraq; 10 Department of Physics, College of Science, University of Kufa, Kufa, Iraq; 11 College of Health and Medical Technology, Al-Ayen University, Thi-Qar, Iraq

**Keywords:** Biochemistry, Biological techniques, Biotechnology, Chemistry, Nanoscience and technology

## Abstract

According to recent studies on the benefits of natural polymer-based hydrogels in biomedical applications, gellan gum (GG)/acacia gum (AG) hydrogel was prepared in this study. In order to regulate the mechanical behavior of the hydrogel, graphite carbon nitride (g-C_3_N_4_) was included in the hydrogel matrix. In addition, metal oxide nanoparticles ZnCuFe_2_O_4_ were added to the composite for antibacterial activity. The prepared GG–AG hydrogel/g-C_3_N_4_/ZnCuFe_2_O_4_ nanobiocomposite was characterized by using FE-SEM, FTIR, EDX, XRD and TGA. The nanobiocomposite exhibited spherical morphology, which was related to the incorporation of the metal oxide nanoparticles. GG–AG hydrogel/g-C_3_N_4_/ZnCuFe_2_O_4_ nanobiocomposite showed 95.11%, 92.73% and 88.97% biocompatibility toward HEK293T cell lines within 24 h, 48 h and 72 h incubation, respectively, which indicates that this nanobiocomposite is completely biocompatible with healthy cells. Also, the nanobiocomposite was able to inhibit *Pseudomonas aeruginosa* biofilm growth on its surface up to 87%. Rheological studies showed that the nanobiocomposite has a viscoelastic structure and has a water uptake ratio of 93.2%. In comparison with other similar studies, this nanobiocomposite has exhibited superior antibacterial activity complete biocompatibility and proper mechanical properties, high swelling and water absorption capability. These results indicate that GG–AG hydrogel/g-C_3_N_4_/ZnCuFe_2_O_4_ nanocomposite can be considered as a potential candidate for biomedical applications such as tissue engineering and wound healing.

## Introduction

Recently, much research has been devoted to the unique three-dimensional structures of hydrogels and their use as a base component in composites^1^. Hydrogels have been widely used in non-biomedical applications such as catalysis^2^ and biomedical applications such as wound healing^3^, tissue engineering^4^, protein and gene delivery^5,6^, and drug delivery^7^ due to their swelling capacity, water and cargo absorbing ability, adjustable mechanical properties and mesoporous structure with tunable physicochemical properties. Hydrogels are created when cross-linked bonds are formed between two polymeric chains which could be equal or not equal^8^. These polymeric chains can have either natural or synthetic sources. However, polymers with natural bases are known for being biodegradable, biocompatible, naturally abundant and environment friendly^9,10^. Therefore, natural polymer-based hydrogels have been significantly studied in biomedical applications due to their biofriendly nature and low toxicity^11,12^. Many different natural polymers such as Chitosan^13^, Guar Gum^14^, pectin^15^, cellulose^16^, lignin^17^, Xanthan Gum^18,19^ and agar^20^ have been for preparation of nature hydrogels. One of the natural polymers that have been investigated for the preparation of natural-based hydrogels is gellan gum (GG)^21,22^. GG is a biodegradable and biocompatible linier polysaccharide with chains consisting of tetrasaccharide repeating units of L-rhamnose, D-galactose and D-glucuronate^23^. Hydrogels structures can be formed with the incorporation of two different types of polymeric chains. The second polymer can be either natural or synthetic, considering the desired application^24^. As another natural polymer, acacia gum (AG) has been widely studied for biomedical applications^25^. Hydrogels containing AG have been studied for wound healing applications due to their water absorbing capacity and potential antioxidant properties^26^. In order to adjust the mechanical properties of the hydrogel for the desired application in different biological or non-biological environments, different components are used^27,28^. Among these components, carbon-based nanomaterials such as graphene oxide, and carbon hybrids such as graphitic carbon nitride (g-C_3_N_4_) have been used in many researches^29^. g-C_3_N_4_ exhibits a mild antibiotic activity witch can act synergistically with the other antimicrobial agents in the hydrogel structure^30^. In addition, in order for the hydrogel to exhibit a suitable biocidal or anticancer property in a biological environment, metal-based nanoparticles such as metal oxides can be used^31,32^. Metal oxides and their composites can exhibit photocatalytic property, antibacterial and anticancer activity^33^. Other than biomedical applications, metal oxides exhibit high potentials for non-biomedical applications such as designing high performance supercapacitors^34^. The hydrogel structure can also reduce the cytotoxicity of metal oxides, making them more suitable for biomedical applications^35^. Metallic nanoparticles containing metals with biocidal properties such as Zn, Cu, Pd or Ag have proven to be beneficial in reducing infection and accelerate healing process in a biological environment^32,36,37^. Considering the significant advantages of metal oxide nanoparticles in various biomedical and non-biomedical applications, many studies have been devoted to the development of newer and greener methods for their preparation^38,39^.

In this study, a natural-based hydrogel was prepared by cross-linking GG and AG polymeric chains using ZnCl_2_ inorganic cross-linking agent. This inorganic cross-linker, can also exhibit a mild synergic biocidal activity due to the presence of the element Zn, which is an antibacterial metal. Then, the prepared GG–AG hydrogel was mixed with g-C_3_N_4_ to improve the mechanical behavior of the nanobiocomposite. Afterwards, the GG–AG hydrogel/g-C_3_N_4_ was mixed with ZnCuFe_2_O_4_ metal oxide nanoparticles with antibacterial properties. The final obtained GG–AG hydrogel/ g-C_3_N_4_/ ZnCuFe_2_O_4_ nanobiocomposite was subjected to FE-SEM, FTIR, EDX, XRD and TGA characteristic analyses. In addition, in order to evaluate the behavior of nanobiocomposite in a biological environment, it was subjected to MTT test towards healthy cell lines and antibacterial test to determine the biocidal property of GG–AG hydrogel/g-C_3_N_4_/ZnCuFe_2_O_4_. Finally mechanical properties of the nanobiocomposite were assessed. Test results exhibited that GG–AG hydrogel/g-C_3_N_4_/ZnCuFe_2_O_4_ shows 95.11%, 92.73% and 88.97% cell viability within 24 h, 48 h and 72 h experiment on HEK293T (human embryonic kidney cell lines), respectively. Also, *P. aeruginosa* biofilm growth was inhibited up to 87%, which was remarkable. In addition, rheological studies indicated the formation of a viscoelastic structure.

**Figure 1:**
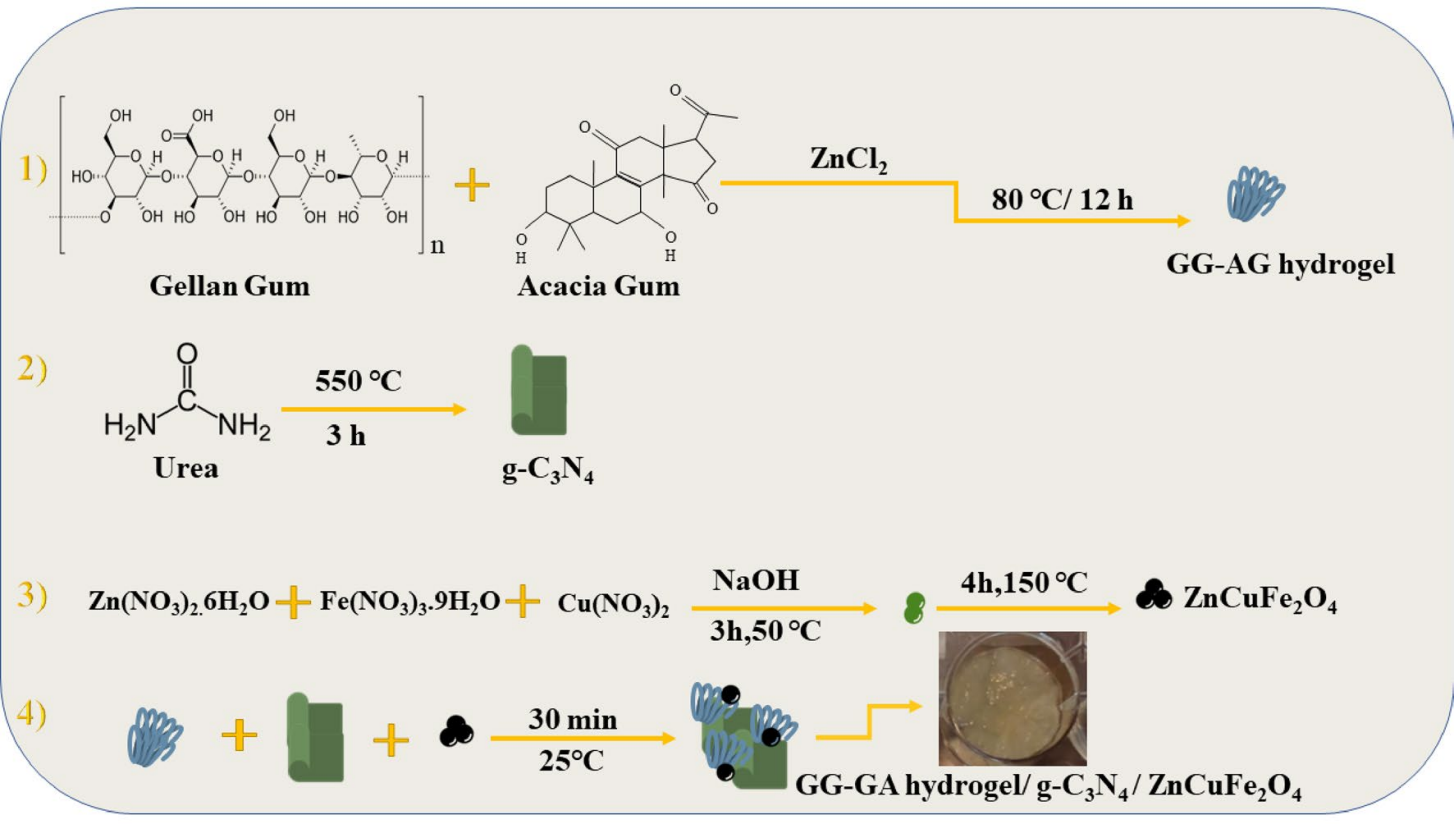
Schematic illustration of the preparation steps of the GG–AG hydrogel/g-C_3_N_4_/ZnCuFe_2_O_4_.

The most important factors in designing a nanobiocomposite for tissue engineering and wound healing are low toxicity toward healthy cells, proper mechanical properties and sufficient antibacterial activity and infection control ability. The designed nanobiocomposite in this study, exhibited low cytotoxicity toward HEK293T healthy cell lines due to its natural-based hydrogel, suitable mechanical properties due to the addition of g-C_3_N_4_ and remarkable antibacterial activity due to the synergistic antibacterial effects of the g-C_3_N_4_ and ZnCuFe_2_O_4_ metal oxide nanoparticles. These results indicates that this nanobiocomposite can be considered as a good potential candidate for tissue engineering and wound healing applications (Fig. 1).

## Materials and methods

### Materials

All materials used in this study including GG and AG polymers, urea, ZnCl_2_, Zn(NO_3_)_2_·6H_2_O, Fe(NO_3_)_3_·9H_2_O, Cu(NO_3_)_3_ and NaOH were purchased from Merk, Sigma-Aldrich and Fluka companies.

### Methods

#### GG–AG hydrogel preparation

0.5 g of GG powder was dispersed in 50 mL distilled water in a 200 mL beaker at room temperature. Separately, 0.5 g AG powder was dispersed in 50 mL distilled water and added to the GG solution. The mixture was stirred continuously at 70–80 °C for 30 min and then, ZnCl_2_ was dissolved in 20 mL of distilled water, sonicated for 30 s and added to the above mixture. After 12 h, the produced hydrogel was collected, cooled at room temperature and freeze dried for analyses.

#### g-C_3_N_4_ preparation and GG–AG hydrogel/ g-C_3_N_4_ composite preparation

g-C_3_N_4_ was prepared based on previously reported methods^40^. Firstly, urea was dried for 24 h at 80 °C oven and was heated at 550 °C oven for 3 h in a covered crucible. The yellow powder of g-C_3_N_4_ was collected after the mentioned time. 5 mL of the prepared GG–AG hydrogel was mixed with 0.02 g g-C_3_N_4_ powder and was stirred continuously at room temperature for 30 min. 5 mL of the prepared composite was collected for the next step and the rest of the composite was collected and freeze dried for analyses.

#### ZnCuFe_2_O_4_ preparation and GG–AG hydrogel/ g-C_3_N_4_/ ZnCuFe_2_O_4_ nanobiocomposite formation

1 g Zn(NO_3_)_2_·6H_2_O, 1.3 g Fe(NO_3_)_3_·9H_2_O and 1 g Cu(NO_3_)_2_ were mixed and added to 100 mL distilled water and sonicated for 30 s. Afterwards the mixture was stirred at room temperature for 20 min. 0.5 g NaOH was dissolved in 10 mL distilled water and added to the above mixture. The solution was stirred at 50 °C for 3 h and after the mentioned time, the mixture was kept in 150 °C oven for 4 h in a stainless-steel autoclave. Afterwards, it was washed with distilled water and ethanol for several times, centrifuged and dried at 60 °C oven overnight. 0.02 g of the prepared ZnCuFe_2_O_4_ was added to 5 mL GG–AG hydrogel/g-C_3_N_4_ composite and stirred at room temperature for 30 min. The final composite was collected and freeze dried for further investigations.

#### Thermogravimetric analysis (TGA)

TG analysis was performed using Bahr-STA 504 instrument (Germany). In order to perform the TGA, 5.0 mg of the sample was transferred into alumina pans under argon atmosphere and 1 L/h flow rate. Heating range was between 50 and 600 °C with 10 °C/min rate.

#### Energy-dispersed X-ray spectroscopy (EDX)

The elemental composition of the nanobiocomposite was determined by EDX analysis (SAMx model, France) with ultrathin window detector.

#### Field-emission scanning microscopy (FE-SEM)

For morphological identification of the samples, they were subjected to FE-SEM analysis (ZEISS-Sigma VP model, Germany) at 15 kV. Samples were mounted on stainless-steel stub by double side carbon tape (Agar Sputter Coater model, Agar scientific, England).

#### Fourier-transform infrared spectroscopy (FT-IR)

FT-IR analysis was performed by (Shimadzu FT-8400s model, Japan), by using KBr pellet preparation method. 0.1–1.0% of each sample was mixed with 200–250 mg KBr and the prepared pellets were subjected to FT-IR frequency range of 400–4000 cm^−1^ at 25 °C.

#### X-ray diffraction (XRD)

XRD was performed using PANalytical X-PERT-PRO MPD at 2Ɵ = 5°–85° with STA504 analyzer in the temperature range of 50°–550° and in air (10 °C/min).

#### Nanobiocomposite extraction

The synthesized GO/Casein/LDH/Alg/Fe_3_O_4_ nanobiocomposite was extracted by dispersing 50 mg of it in 1 ml of phosphate buffer saline (PBS) using shaker incubator for 48 h at 37 °C.

#### MTT assay

MTT assay was performed to determine the biocompatibility of nanobiocomposite. For this purpose, HEK293T cells were firstly cultured in DMEM/F12 medium with 10% FBS. Afterwards, 5 × 10^3^ cells/well were moved to 96-well plates and 10 μL of the nanobiocomposite extract was transferred into each cell and incubated for 24 h, 48 h and 72 h. PBS-treated cells were also considered as negative control. Afterwards, cells were treated with MTT (3-4,5dimethylthiazol-2-yl)-2,5diphenyl tetrazolium bromide) (Sigma, USA) and incubated for another 4h at 37 °C. 1% SDS was added to cell/wells and incubated at 37 °C for 16h. By using a microplate reader spectrometer (BioTek. USA) at 550 nm, optical densities (OD) were measured and cell viability was assessed using following formulas^41^:

$${\text{Toxicity \% }} = \left( {\frac{{{\text{Mean }}\,{\text{OD}}\,{\text{ of}}\,{\text{ sample}}}}{{{\text{Mean }}\,{\text{OD }}\,{\text{of }}\,{\text{control}}}}} \right){ } \times { }100\quad {\text{Viability \% }} = { }100 - {\text{Toxicity \% }}$$

#### Anti-biofilm activity

Antibacterial activity of the prepared nanobiocomposite was studied using tissue culture plate (TCP) anti-biofilm assay. For this purpose, 1 cm^2^ of the nanobiocomposite was sterilized in 70% ethanol alongside with a polystyrene piece as the positive control, and were dried at 37 °C in an incubator. Each piece was then transferred to sterilized tubes containing *Pseudomonas aeruginosa* bacteria (ATCC 27853)with 10^7^ colony-forming unit (CFU)/mL concentration in a culture medium of Nutrient Brot (NB). Afterwards, tubes were incubated for 24 h at 37 °C in a shaker incubator with shake speed 150 rpm. Samples were washed with PBS and stained by 0.1% crystal violet solution for 5 min and then were washed with 33% acetic acid solution. Finally, by using a microplate reader (STAT FAX 2100, BioTek, Winooski, USA), the resulting solution’s OD was evaluated at 570 nm^42^.

#### Statistical analysis

Statistical analysis for the comparison all results was accomplished by a t-test by SPSS Statistics 22.0 software (SPSS Inc. Chicago, IL, USA). The values of* P* $\ge$ 0.05 (*), *P* $\le$ 0.05 (**) and *P* $\le$ 0.001 (***) were considered as statistically insignificant, significant and very significant, respectively.

#### Rheological studies

The samples (hydrogel and final nanobiocomposite) were swollen in ultra-pure water at room temperature for 24 h before the rheological measurements were taken using an RMS/MCR 302 rheometer (Anton-Paar Co., USA) equipped with a 20 mm parallel plate. Measurement of the storage modulus (G′) and loss modulus (G″) were conducted at shear stress range from 0.01 to 1000 Pa at controlled frequency of 0.1 Hz. The measurements were stopped when both G′ and G″ began to decrease notably. For this test, three specimens were measured, and the values were averaged. Dynamical mechanical analysis of the swollen samples was performed by changing the oscillatory stress from 0.01 to 1000 Pa at a constant frequency (1 Hz)^43^.

#### Compressive tests

The compressive mechanical properties of GG–AG hydrogel/g-C_3_N_4_/ZnCuFe_2_O_4_ nanobiocomposite, were measured according to the procedure of Bhardwaj et al. and ASTM method F451-95 with some modifications^43^. This test was performed using a Universal Testing machine (SANTAM, STM-20 model) with a load cell capacity of 0.1 kN with crosshead speed 1 mm/min at room temperature. Based on this method, the specimens with a thickness of 10 mm and a diameter of 13 mm were cut manually from nanobiocomposite using a razor blade. In the following, the samples were soaked in PBS solution for 2 h and this test was accomplished under wet conditions. At least three fragments were tested and the mean values were reported.

#### Swelling properties

Freeze-dried nanobiocomposite was immersed in UPW at 25 °C for 48 h. Surplus UPW was then removed from the surface of sample and the wet weight of the nanobiocomposite was determined. The swelling ratio and the water uptake in the sample were calculated as follows:

$$\begin{aligned} & Swelling \,ratio \left( \frac{g}{g} \right) = \left[ {\frac{Ws - Wd}{{Wd}}} \right] \\ & Water \,uptake \left( \% \right) = \left[ {\frac{Ws - Wd}{{Ws}}} \right] \times 100 \\ \end{aligned}$$

In this formula, W_s_ and W_d_ are the weights of dried and swollen nanobiocomposite, respectively^44^.

#### Biodegradability assay

For degradation experiments, GG–AG hydrogel/g-C_3_N_4_/ZnCuFe_2_O_4_ nanobiocomposite was placed into PBS at pH = 7.4 and 37  °C The buffer solution was refreshed every 3 days. This test was performed up to 10 days and at the selected time points, three samples of nanobiocomposite were removed from the buffer and weighed wet after surface wiping. Afterwards, they were rinsed with UPW and dried in a vacuum oven at 37 °C for 24 h. Water absorption and weight loss were calculated according to these formulas:

$$\begin{aligned} & Water\, absorption \left( \% \right) = \left[ {\frac{Wa - W0}{{W0}}} \right] \\ & Weight \,loss \left( \% \right) = \left[ {\frac{W0 - Wt}{{W0}}} \right] \\ \end{aligned}$$

where W0 is the starting dry weight, Wa is the wet sample weight after removal from the solution, and Wt is the dry sample weight after removal^45^.

## Results and discussion

### FE-SEM and EDX analyses

**Figure 2:**
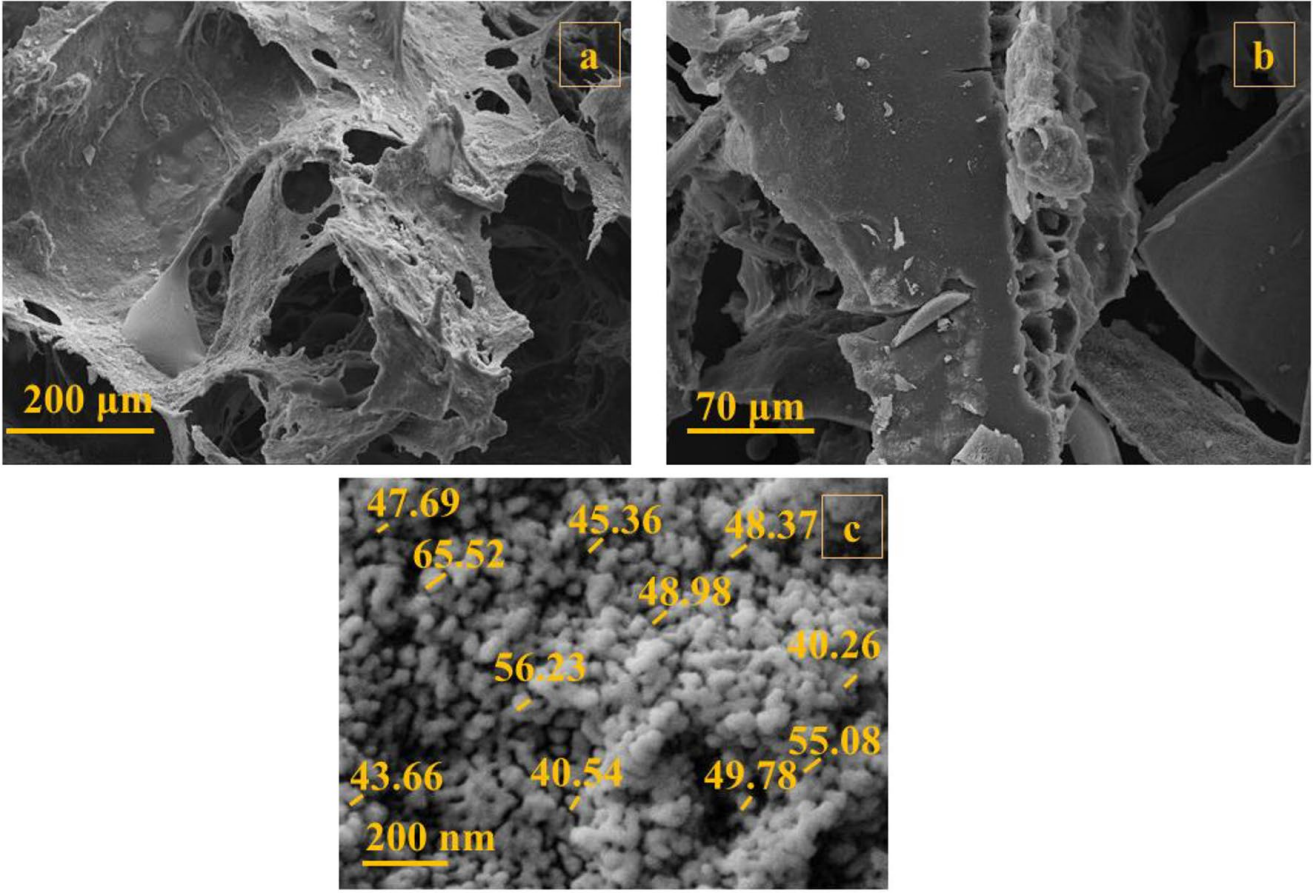
FE-SEM images of (**a**) GG–AG hydrogel, (**b**) GG–AG hydrogel/g-C_3_N_4_ and (**c**) GG–AG hydrogel/g-C_3_N_4_/ZnCuFe_2_O_4_ nanobiocomposite.

**Figure 3:**
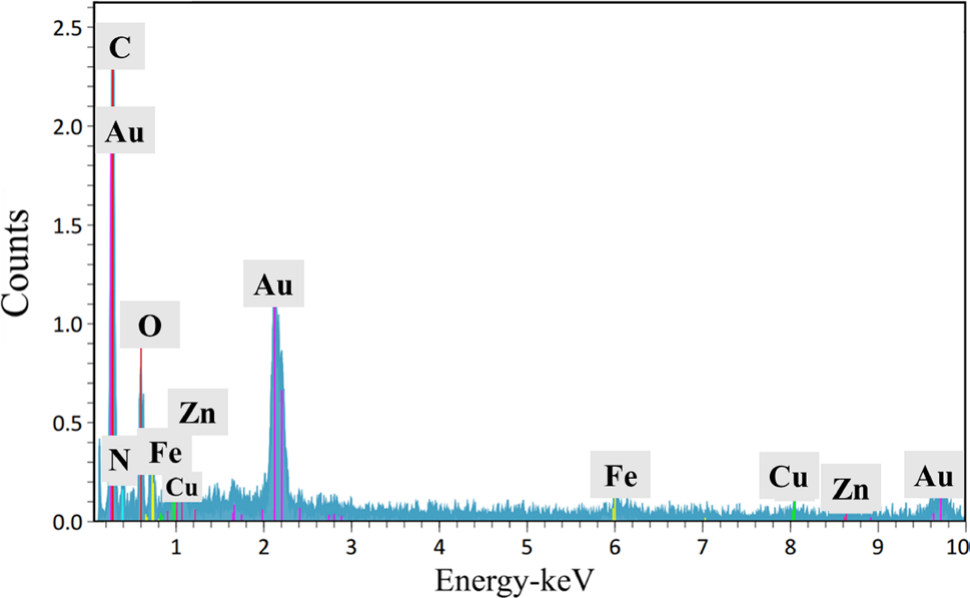
EDX analysis performed on GG–AG hydrogel/g-C_3_N_4_/ZnCuFe_2_O_4_ nanobiocomposite.

The morphological transformations of the composite were detected using FE-SEM imaging in every synthetic step (Fig. 2). As illustrated in (Fig. 2a), GG–AG hydrogel has a porous structure. After the addition of g-C_3_N_4_ (Fig. 2b), the morphological structure seems to be stiffer and rougher and also less mesoporous, which is due to the incorporation of g-C_3_N_4_ into the hydrogel structure^46^. The final synthetic step is illustrated at (Fig. 2c) and is related to the final GG–AG hydrogel/g-C_3_N_4_/ZnCuFe_2_O_4_ nanobiocomposite. After the addition of metal oxide nanoparticles, the morphology was completely changed into spherical nanostructures with an aggregable size distribution, mainly between 40 and 60 nm. For elemental detection of the composite, it was subjected to EDX analysis. Presence of carbon and oxygen is related to the hydrogel structure. Carbon is also present in the g-C_3_N_4_ structure. Presence of nitrogen is attributed to the g-C_3_N_4_ within the nanobiocomposite structure, while zinc, iron and copper are related to the incorporated metal oxide. Presence of gold element is due to the gold covering during the analyze and is not relevant to the nanobiocomposite structure (Fig. 3).

### TGA

**Figure 4:**
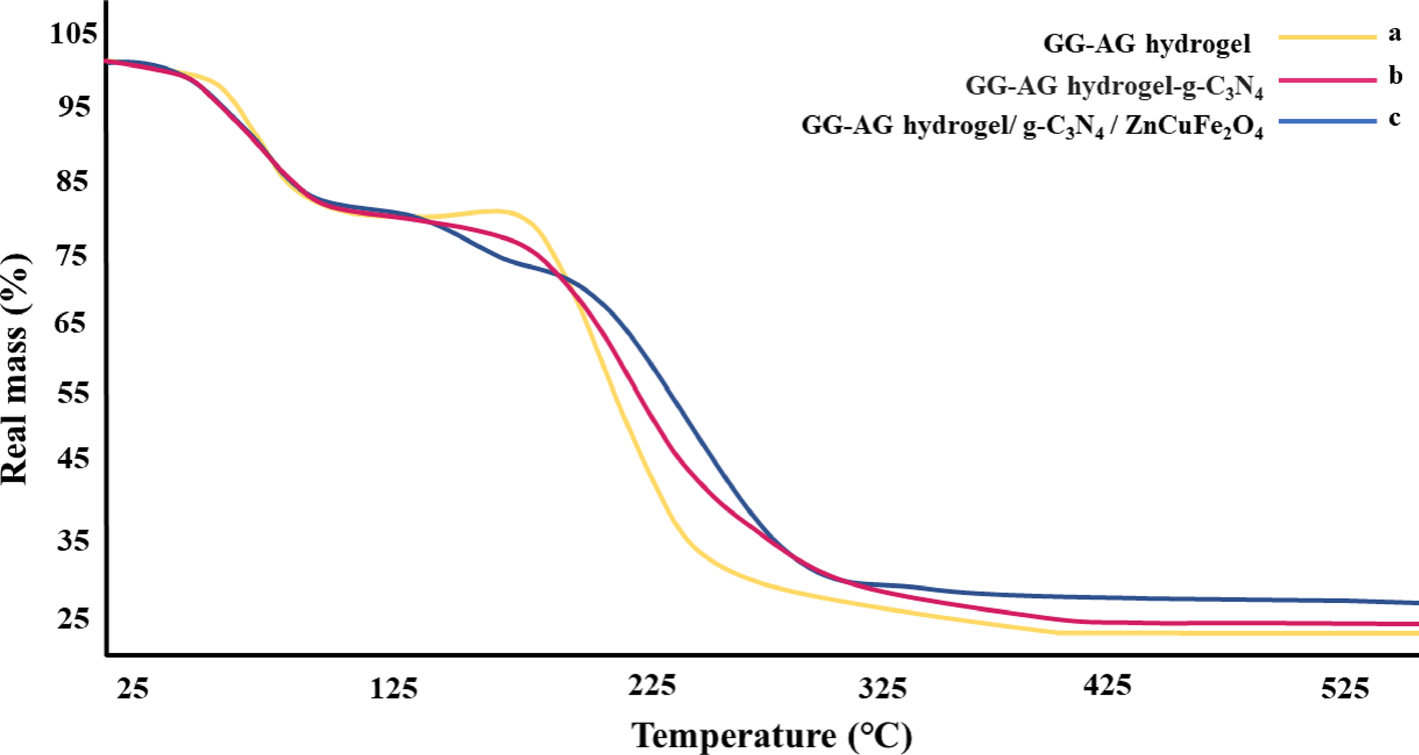
TGA analysis of (**a**) GG–AG hydrogel, (**b**) GG–AG hydrogel/g-C_3_N_4_ and (**c**) GG–AG hydrogel/g-C_3_N_4_/ZnCuFe_2_O_4_ nanobiocomposite.

In order to analyze the composite’s thermal stability, TGA assessment was performed in a thermal range of 25–600 °C (Fig. 4). Figure 4a exhibits GG–AG TGA curve. As illustrated in the figure, the minor weight loss at 120–130 °C is related to the GG crystalline protein melting^47^. Also, the major weight loss with a sharp slop is relevant to the destruction of GG and AG polymeric chains destruction^48^. Figure 4b exhibits the TGA curve of GG–AG-g-C_3_N_4_, it is observed that by addition of g-C_3_N_4,_ the thermal stability has increases and the weight loss slop has been moderated, indicating the polymeric chains slower decomposition. Figure 4c exhibits the nanobiocomposite thermal degradation process. The first observed weight loss at around (50–100 °C) is related to the loss of water molecules trapped inside the structure. The major weight loss at around (200–400 °C) is related to the decomposition of the GG and AG polymeric chains and is similar to the previous studies on the decomposition diagrams of these polymers^49,50^. Around 30% of the composite remains unburned, which is related to the polymer ashes, g-C_3_N_4_ and the metal oxide.

### FTIR analysis

**Figure 5:**
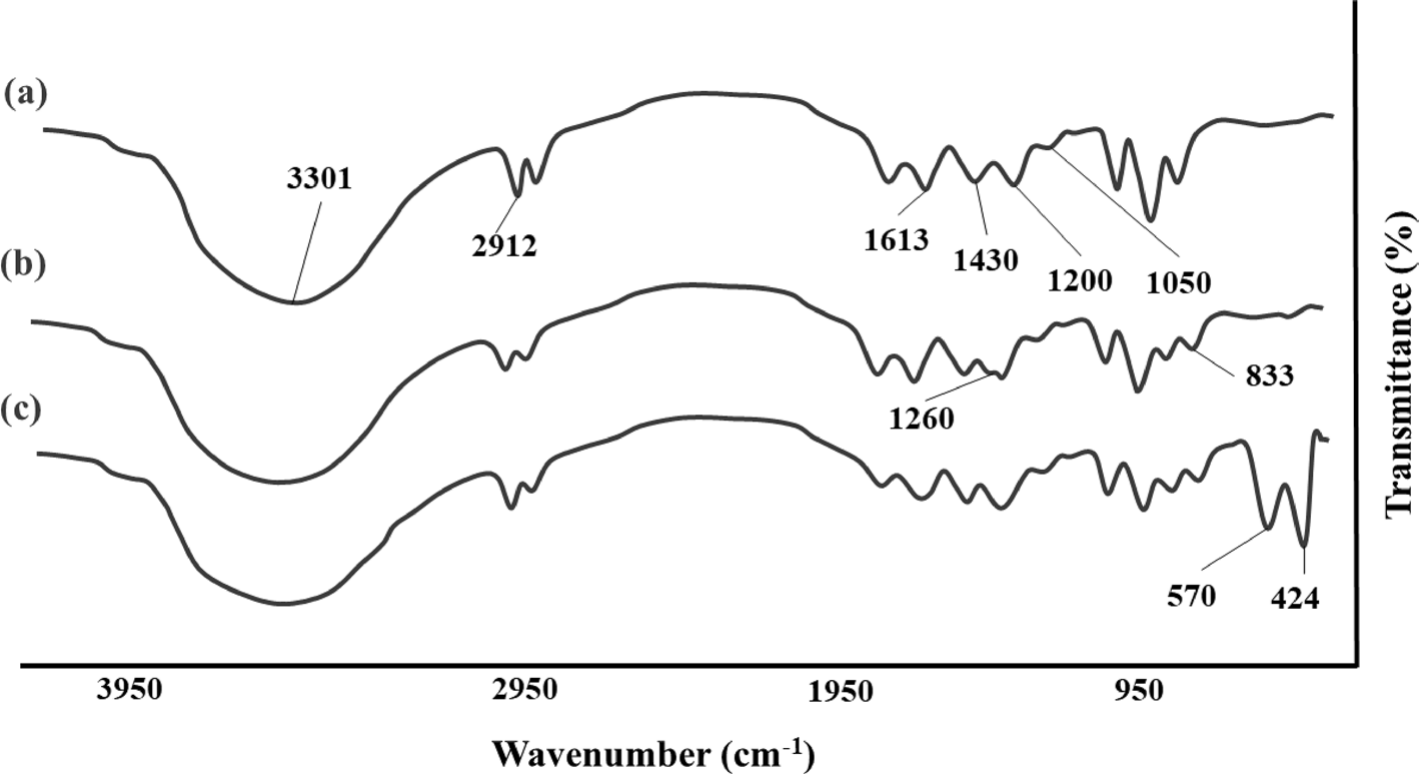
FTIR spectrum of (**a**) GG–AG hydrogel, (**b**) GG–AG hydrogel/g-C_3_N_4_ and (**c**) GG–AG hydrogel/g-C_3_N_4_/ZnCuFe_2_O_4_ composite.

In order to determine each synthetic step’s material incorporation, FTIR analysis was performed in every step. GG-AC spectrum is shown at (Fig. 5a) with a broad absorption band at 3301 cm^−1^, which is relevant to the –OH stretching vibration of polymeric chains. Two peaks at around 2912 cm^−1^, are related to CH_3_ and CH_2_ stretching vibrations of both polymers. Presence of COO^−^ is noticeable by the absorption band at 1613 cm^−1^. Also, peaks at 1200 and 1050 cm^−1^, are relevant to the C–O stretching vibration. Peaks between 1000 and 800 cm^−1^ are related to the ZnCl2 cross-linking agent used in the hydrogel structure^51–53^. As illustrated in (Fig. 5b), g-C_3_N_4_ within the structure is noticed by absorption bands at 1260 cm^−1^ and 833 cm^−1^, which are related to C–N (–C)–C or C–NH–C stretching vibrations and out of plane bending vibration of triazine molecules, respectively^54^. The final synthetic step, GG–AG hydrogel/g-C_3_N_4_/ZnCuFe_2_O_4_ spectrum is exhibited at (Fig. 5c). The spectrum shows peak at under 1000 cm^−1^ with a similar pattern to the previously studies on this metal ferrite. Also, peaks at 570 cm^−1^ and 424 cm^−1^ are related to stretching vibration of (Fe^3+^−O^2−^) tetrahedral and octahedral complexes, respectively^55^.

### XRD

**Figure 6:**
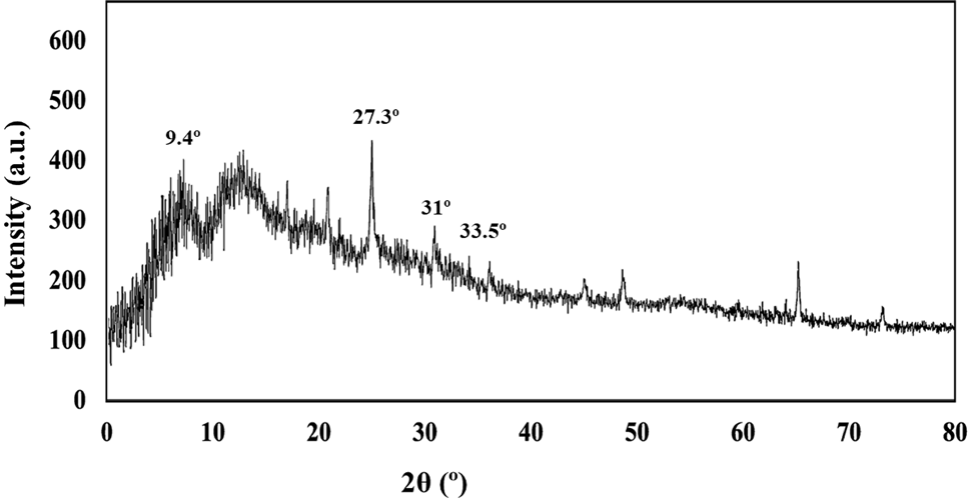
XRD pattern of the GG–AG hydrogel/g-C_3_N_4_/ZnCuFe_2_O_4_ nanocomposite.

Based on the previous studies on ZnCuFe_2_O_4_ XRD pattern^56^, the presence of nanoparticles was detected based on JCPDS data file No. 25-0283 (CuFe2O4) and No. 22-1012 (ZnFe2O4) as plane reflections for the cubic spinal phase. Peaks at around 2θ = 31° and 33.5°, which were accorded to (2 2 0) and (3 1 1) panes, respectively, were identified as the cubic spinal phase of ZnCuFe_2_O_4_ structure^56^. Also, due to the hydrogel structure of the composite, the pattern appears to be amorphous rather than crystalline. Also, the successful incorporation of the g-C_3_N_4_ within the composite structure is observed in the XRD pattern with characteristic peaks at around 9.4° and 27.3° which were accorded to (1 0 0) and (0 0 2) plane of g-C_3_N_4_^57,58^ (Fig. 6).

### Biocompatibility

**Figure 7:**
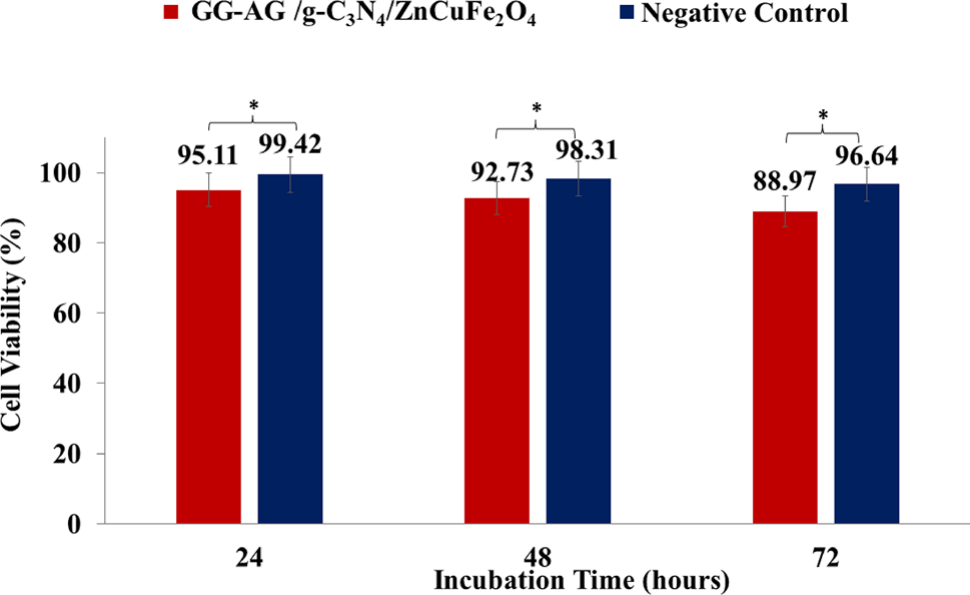
Histogram of the cell viability percentage after different incubation times of GG–AG hydrogel/g-C_3_N_4_/ZnCuFe_2_O_4_ nanobiocomposite extract and PBS as negative control (* = insignificant, *P* ≥ 0.05).

As shown in Fig. 7, the viability percentages of HEK293T cells treated with GG–AG hydrogel/g-C_3_N_4_/ZnCuFe_2_O_4_ nanobiocomposite extract, after 24 and 48 h of incubation were 95.11% and 92.73%, respectively. This value decreased to 88.97% after 72 h of incubation. Results are the average of three independent experiments. These results illustrate that this nanobiocomposite was biocompatible with HEK293T cells and in comparison with pervious studies on hydrogel-based composites, the prepared nanobiocomposite exhibited extremely high biocompatibility and biosafety and shows high potential in biomedical fields^59,60^.

### Anti-biofilm activity

**Figure 8:**
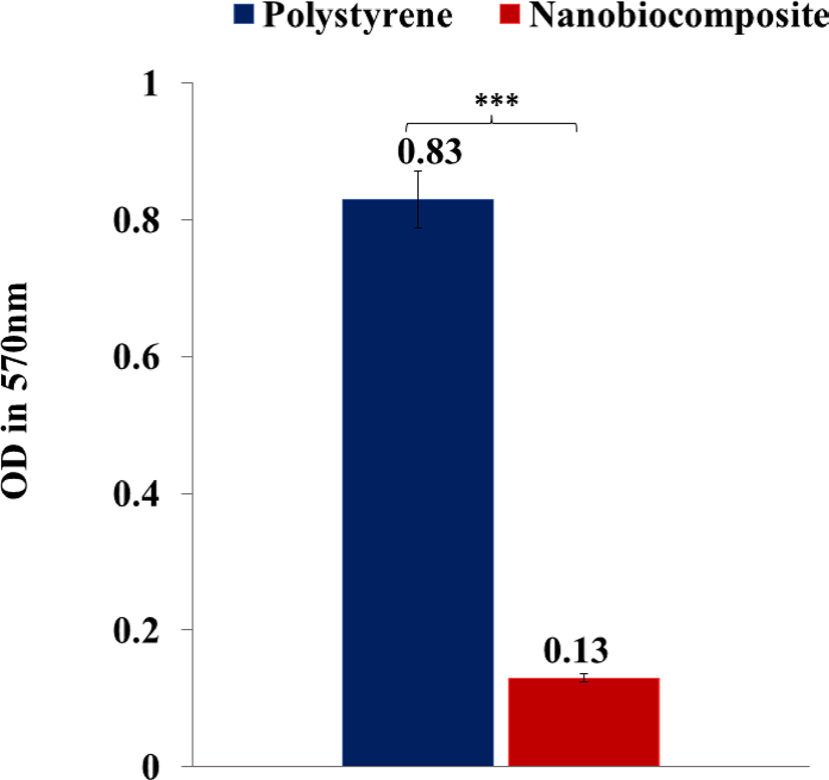
Anti-biofilm histogram of polystyrene and GG–AG hydrogel/g-C_3_N_4_/ZnCuFe_2_O_4_ nanobiocomposite pieces (*** = very significant, *P* $\le$ 0.001).

**Figure 9:**
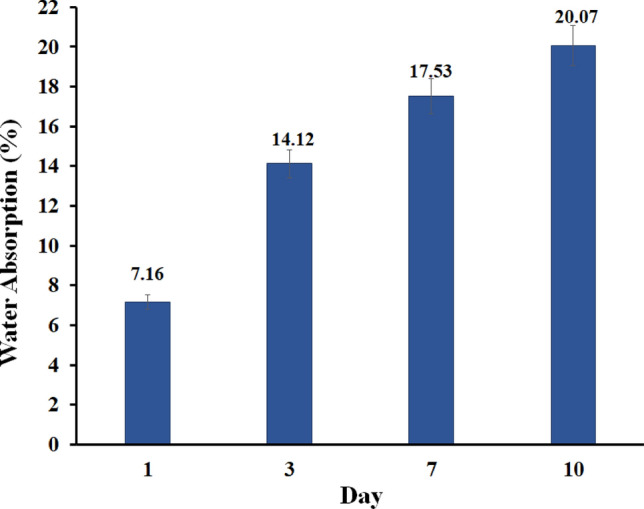
Water absorption of the GG–AG hydrogel/g-C_3_N_4_/ZnCuFe_2_O_4_ nanobiocomposite soaked in PBS for various periods.

As demonstrated, after washing the biofilms from the pieces, the absorbance of the resulting solutions was measured at 570 nm in the 96 micro-well plate. As can be seen in Fig. 9, this value for polystyrene and nanobiocomposite pieces was 0.83 and 0.13, respectively. According to the statistical analysis, the anti-biofilm activity of the GG–AG hydrogel/g-C_3_N_4_/ZnCuFe_2_O_4_ fragment was very significantly (*P* $\le$ 0.001) higher than that of the polystyrene piece. These results indicates that the nanobiocomposite was able to inhibit *P. aeruginosa* biofilm formation on its surface up to 87% and has a considerable anti-biofilm activity as shown in Fig. 8.

### Rheological studies

The analysis provided information about the storage modulus (G′), the loss modulus (G′′) and the phase angle (Table 1). The phase angle of 0° and 90° indicates a perfectly elastic material and viscous material, respectively. Also, a larger value of G′ when compared to G′′ indicates that the analyzed material has pronounced elastic properties. For GG–AG hydrogel/g-C_3_N_4_/ZnCuFe_2_O_4_ nanobiocomposite it was found that the storage modulus was higher than the loss modulus (G′/G′′ > 1), exhibiting the formation of an elastic network.

**Table 1:** Mechanical behavior of the GG–AG hydrogel compared to the final nanobiocomposite.

	G′ (Pa)	G′′ (Pa)	Phase angle (°)	Max. oscillation stress (Pa)
GG–AG hydrogel	2472 (± 65.4)	1123.4 (± 6.1)	18.9 (± 3.1)	4.7 (± 0.0)–6.8 (± 0.0)
Nanobiocomposite	5731 (± 103.2)	1689.9 (± 13.1)	22.7 (± 6.3)	7.8 (± 0.0)–11.3 (± 0.0)

### Compressive tests

The compressive strength, was calculated using the following formula:

$$\sigma = {\raise0.7ex\hbox{$F$} \!\mathord{\left/ {\vphantom {F A}}\right.\kern-0pt} \!\lower0.7ex\hbox{$A$}}$$

where F is force and A is area defined as the cross section of the sample^61^. Accordingly, the compressive strength of the GG–AG hydrogel/g-C_3_N_4_/ZnCuFe_2_O_4_ nanobiocomposite was obtained, the results of which are visible in Table 2.

**Table 2:** The compressive strength of synthesized GG–AG hydrogel/g-C_3_N_4_/ZnCuFe_2_O_4_ nanobiocomposite.

Sample	Compressive strength (kPa)
GG–AG hydrogel/g-C_3_N_4_/ZnCuFe_2_O_4_	711.69

### Swelling properties

The water-binding ability was measured using the swelling ratio and water uptake. The swelling ratio and water uptake of the GG–AG hydrogel/g-C_3_N_4_/ZnCuFe_2_O_4_ nanobiocomposite were bout 8 and 93.2%, respectively, which is acceptable compare to other studies^46^.

### Biodegradability assay

**Figure 10:**
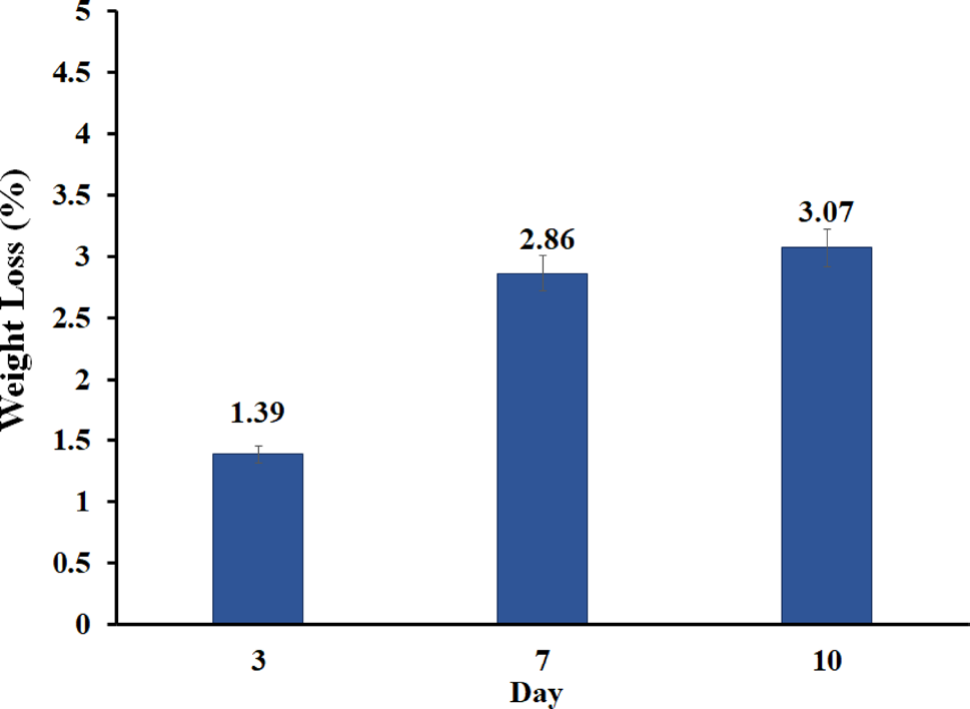
Weight loss of the GG–AG hydrogel/g-C_3_N_4_/ZnCuFe_2_O_4_ nanobiocomposite soaked in PBS in several time periods.

Figures 9 and 10 show the water absorption and weight loss of GG–AG hydrogel/g-C_3_N_4_/ZnCuFe_2_O_4_ nanobiocomposite soaked in PBS for various periods. Water absorption of the nanobiocomposite increased throughout the entire incubation period. Also, weight loss of nanobiocomposite occurred very slowly, without appreciable weight change throughout the degradation period.

## Conclusions

Recently, much attention has been attributed to natural polymer-based hydrogels and their applications in biomedical fields due to their biocompatibility, biodegradability, natural abundance and adjustable mechanical properties. Thus, in this study, two of the most frequently used natural polymers, GG and AG were used to prepare the GG–AG hydrogel. Afterwards, in order to increase the mechanical behavior of the hydrogel and also for better antibacterial performance, the GG–AG hydrogel was incorporated with g-C_3_N_4_ and ZnCuFe_2_O_4_ nanoparticles and the final GG–AG hydrogel/g-C_3_N_4_/ZnCuFe_2_O_4_ nanobiocomposite was established. The GG–AG hydrogel/g-C_3_N_4_/ZnCuFe_2_O_4_ nanobiocomposite was characterized using FT-IR, FE-SEM, EDX, TGA and XRD analyses. Moreover, the GG–AG hydrogel/g-C_3_N_4_/ZnCuFe_2_O_4_ nanobiocomposite was able to inhibit the growth of *P. aeruginosa* biofilm on its surface up to 87%. Biocompatibility evaluations showed that the nanobiocomposite showed 95.11%, 92.73% and 88.97% to HEK293T cells biocompatibility within 24 h, 48 and 72 h, respectively, and was completely biocompatible. Also, rheological studies showed the formation of a viscoelastic structure with 93.2% water uptake capacity ratio. These results indicate that the GG–AG hydrogel/g-C_3_N_4_/ZnCuFe_2_O_4_ nanobiocomposite can be considered as an excellent potential candidate for biomedical applications, including wound healing and tissue engineering.

## Data Availability

All data generated or analyzed during this study are included in this published article.
